# Small bowel obstruction by a giant fecal bezoar in a blind loop of small intestine: a case report

**DOI:** 10.3389/fmed.2025.1644026

**Published:** 2025-09-10

**Authors:** Ziwen Chen, Youbo Wu, Kexing Xi

**Affiliations:** ^1^Department of Gastrointestinal Hernia Surgery, Ganzhou Hospital-Nanfang Hospital, Southern Medical University, Ganzhou, China; ^2^Medical College of Jiaying University, Meizhou, China; ^3^Cancer Center, Beijing Tsinghua Changgung Hospital, School of Clinical Medicine, Tsinghua University, Beijing, China

**Keywords:** blind loop syndrome, fecalith, intestinal obstruction, surgical technique, side-to-side anastomosis

## Abstract

Blind loop syndrome (BLS) is a clinical condition characterized by bacterial overgrowth and stasis within intestinal blind loops, which may result from anatomical abnormalities such as diverticula, fistulae, or surgical anastomoses. While end-to-side and side-to-side intestinal anastomoses are common surgical techniques, the latter has been associated with a higher risk of BLS due to the potential formation of stagnant segments. This case report presents a rare instance of small intestinal obstruction caused by large fecalith formation within a blind loop 1 year after side-to-side anastomosis. The clinical presentation, diagnostic approach, and management strategies are discussed in detail. This report highlights the importance of surgical technique selection and standardization in preventing BLS-related complications, providing valuable insights for clinical practice.

## Introduction

Blind loop syndrome (BLS), also known as stagnant loop syndrome, is a malabsorption disorder caused by bacterial overgrowth within stagnant intestinal segments (1). These blind loops often result from surgical anastomoses (e.g., side-to-side or end-to-side), congenital anomalies (e.g., diverticula), or pathological fistulae. The syndrome primarily affects patients with a history of abdominal surgery, particularly those involving intestinal reconstruction (2).

Anatomically, BLS most frequently involves the small intestine, especially the jejunum and ileum, where altered motility and luminal stasis promote bacterial proliferation. Clinically, patients present with chronic diarrhea, weight loss, vitamin B12 deficiency, and, in severe cases, obstructive symptoms due to fecalith formation—a rare but critical complication (3).

Diagnosis relies on a combination of imaging (CT enterography, small bowel series) and functional tests (hydrogen breath testing for bacterial overgrowth). Treatment strategies include surgical resection of blind loops, antibiotic therapy, and nutritional support. This case report presents a rare instance of small intestinal obstruction caused by large fecalith formation within a blind loop 1 year after side-to-side anastomosis.

### Case report

A 77-year-old man was admitted to the hospital presenting with a 3-day history of abdominal pain and distension, accompanied by absolute constipation and bilious vomiting. The abdominal pain was primarily characterized by paroxysmal periumbilical pain, without accompanying fever, headache, dizziness, or other symptoms. The patient, originally from a rural area, maintains a diet predominantly featuring fiber-rich foods such as vegetables, consuming approximately 250 g daily, with occasional intake of persimmons and similar fruits. He had a history of undergoing partial small bowel resection with side-to-side anastomosis 1 year prior to admission.

**Figure 1:**
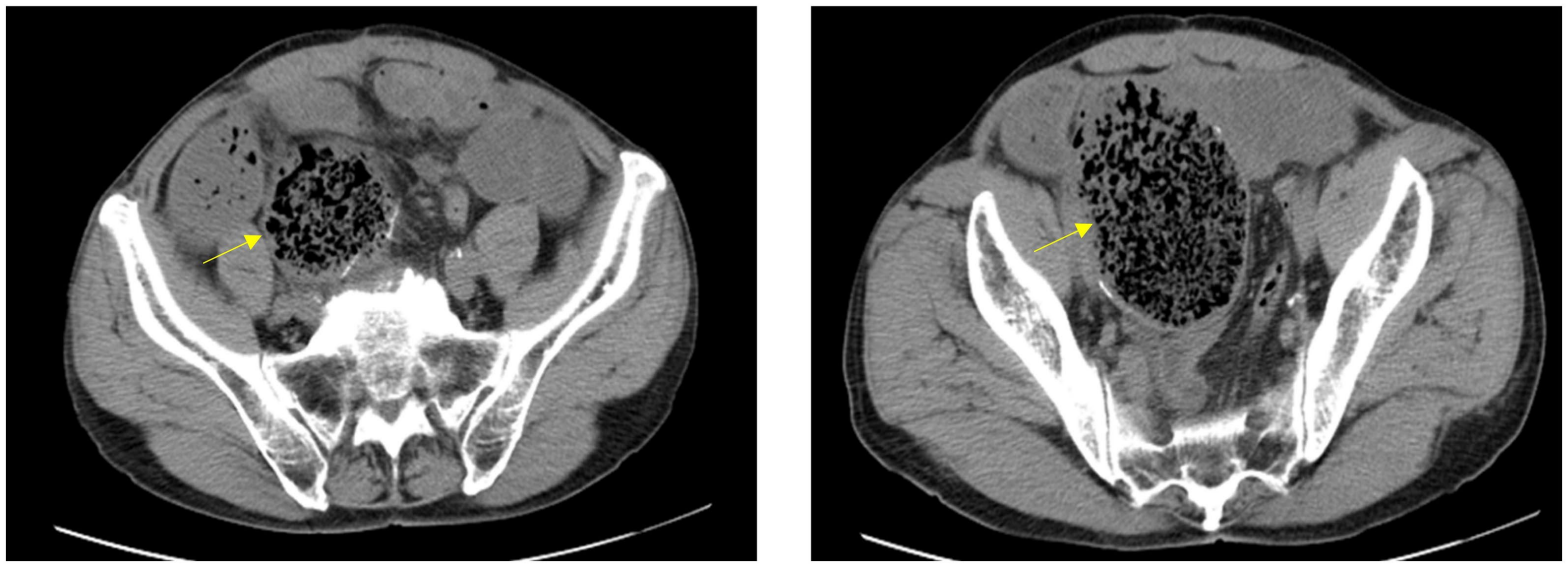
CT scan reveals significant sac-like dilation of the original small intestinal anastomotic blind end, with fecal stagnation forming a mass inside. The proximal intestinal segment shows marked dilation.

Physical examination revealed significant abdominal distension with a palpable mobile mass in the right lower quadrant. There was mild abdominal tenderness but no rebound. A mobile right lower quadrant mass was felt, and bowel sounds were markedly diminished. The patient underwent a comprehensive series of laboratory tests and diagnostic examinations upon hospital admission. The complete blood count revealed a neutrophil count of 7.21 × 10^9/L, with neutrophils accounting for 76.0% of white blood cells. The total white blood cell count was within the normal range. CT scan revealed a bag-like dilatation of the blind end of the original anastomosis, containing a fecal mass and causing intestinal obstruction with a proximal bowel dilatation (Figure 1).

**Figure 2:**
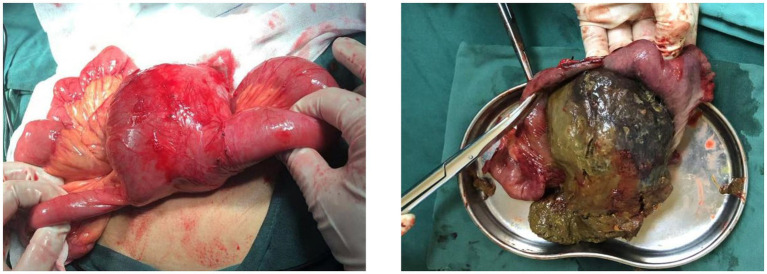
Large cystic solid mass was visible at the blind end of the original small bowel anastomosis. A large fecal bezoar was palpable in the mass.

**Figure 3:**
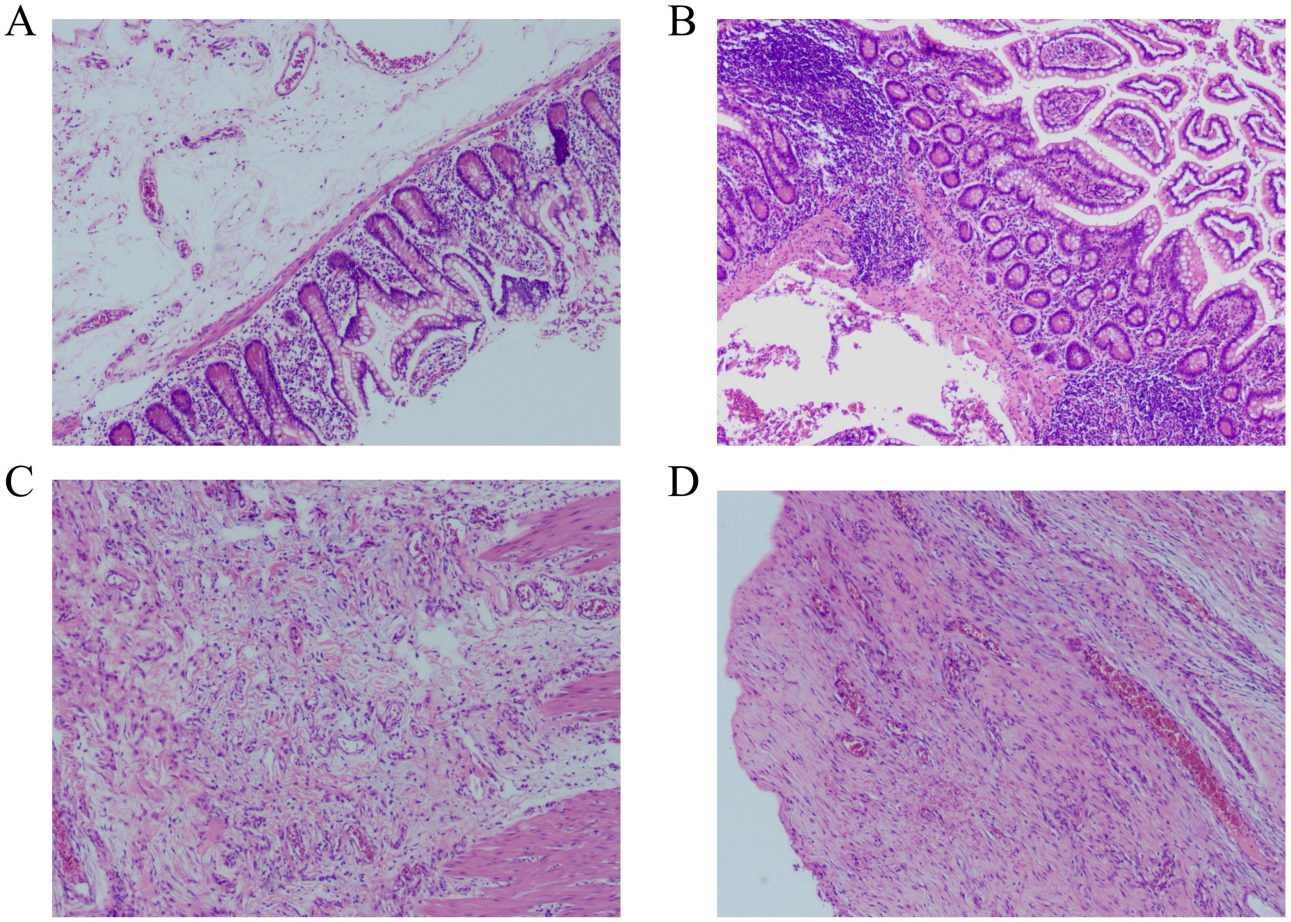
Histopathological examination revealed numerous dilated and congested blood vessels in the submucosal and serosal layers of the intestine, with focal areas demonstrating inflammatory cell infiltration (Hematoxylin–eosin staining). **(A,B)** magnification×40. **(C,D)** magnification×100.

After obtaining consent from the patient and family members, the patient underwent surgical treatment. Emergency exploration revealed the original small bowel anastomosis at about 50 cm from the ileocecal valve with a large fecoloma obstructing the bowel with proximal bowel dilatation and distal collapse (Figure 2). The original anastomosis and the blind end of the anastomosis (including the fecal bezoar) were surgically resected, bowel continuity was restored by performing a sutured end-to-end anastomosis, and the patient was safely discharged from the hospital 7 days after the operation. Postoperative pathological examination revealed an intact full-thickness intestinal wall structure with regularly arranged columnar mucosal epithelium. The submucosal and serosal layers exhibited numerous dilated and congested blood vessels. Focal areas showed inflammatory cell infiltration, consistent with post-obstructive changes in the intestine (Figure 3).

## Discussion

Blind loop syndrome (BLS), also known as stagnant loop syndrome, occurs due to various causes. These include intestinal diverticula, intestinal fistula, or surgery. Such conditions lead to the formation of blind loops within the intestinal canal. Contents stagnate within these blind loops, promoting excessive bacterial colonization. This results in clinical symptoms including diarrhea, steatorrhea, nutritional absorption disorders, and vitamin B12 deficiency-related macrocytic anemia (4–6). Numerous studies have suggested that the primary cause of blind loop syndrome is bacterial overgrowth, leading to intestinal dysbiosis, which subsequently results in the corresponding clinical symptoms (2).

BLS typically arises after surgical procedures (e.g., side-to-side anastomosis and Roux-en-Y reconstruction) or in congenital/acquired strictures, leading to a stagnant loop where bacterial proliferation alters bile salt metabolism and nutrient absorption (7). BLS predominantly affects patients with prior abdominal surgeries, particularly those involving intestinal bypass or blind pouch creation. Less commonly, it occurs in Crohn’s disease, radiation enteritis, or motility disorders (8). In this case, the formation of a fecal bezoar in the blind loop is clinically extremely rare. Our literature search revealed no similar reported cases in the literature.

Diagnosis relies on clinical suspicion, imaging (CT/MRI demonstrating blind loop dilatation ± fecal bezoar), and hydrogen breath testing for bacterial overgrowth (9). CT findings in this case revealed significant pouch-like dilation of the blind end at the original small bowel anastomosis, along with marked proximal intestinal dilation. Differential diagnoses include tumor or foreign body obstruction, necessitating a comprehensive evaluation. Treatment involves surgical resection of the blind loop (if feasible) and fecal bezoar removal, alongside antibiotics (e.g., rifaximin) for bacterial overgrowth (10). Endoscopic fragmentation may be attempted for small fecal bezoars, but laparotomy remains definitive for giant obstructions, as in our case (11). Long-term prevention includes dietary modifications (low-residue diets) and prokinetics for selected patients (12).

The etiology of this case is summarized as follows: First, the side-to-side anastomosis of the small intestine forms a blind loop, causing stagnation of food. Second, the side-to-side anastomosis caused retrograde peristalsis in the distal bowel. This reverse movement dynamically forced intestinal contents into blind loops. Consequently, the contents oscillated, accumulated, and ultimately stagnated within these loops. Third, the patient may have eaten foods that are prone to forming fecaliths, such as persimmons, hawthorns, and black jujubes that are rich in tannins, which can form insoluble fecaliths when encountering gastric acid. When these foods encounter gastric acid, they form water-insoluble ellagic acid protein, which can form lumpy fecal bezoars by gluing together the pulp, plant fiber, etc. (13).

Side-to-side anastomosis carries a higher small bowel obstruction (SBO) risk due to potential blind loop formation and retrograde peristalsis (14). End-to-end anastomosis shows lower obstruction rates but may cause strictures. Functional end-to-side techniques demonstrate intermediate risk. For small bowel obstruction without peritonitis, intestinal necrosis, or ischemia, initial non-surgical management is recommended as the primary approach, particularly in patients with comorbidities affecting vital organs, immunodeficiency, or those at elevated risk of surgical complications, where conservative treatment is frequently favored (15–17). Currently, robust evidence to define the optimal duration of non-surgical therapy is lacking; however, expert consensus supports a period of 3 to 5 days as safe and appropriate, with delays in surgical intervention potentially increasing mortality rates (14, 16, 18–21). For patients with small bowel obstruction who fail conservative management and present with hemodynamic instability, intestinal necrosis, or severe intra-abdominal infection, ‌prompt surgical intervention is strongly indicated (22).

Clinically, many gastrointestinal surgeries can create artificial blind loops. These blind loops may subsequently cause blind loop syndrome (BLS). Alternatively, blind loops can lead to the formation of blind loop fecal bezoars, as described previously. Surgeons should choose an anastomosis that conforms to the physiological structure of the patient and the direction of peristalsis and try not to cause blind loops or blind pouches. If it is necessary to perform an end-to-side or side-to-side anastomosis, surgeons should try to avoid the blind end being too long to reduce the incidence of blind-loop syndrome.

## Conclusion

This case report presents a rare instance of small intestinal obstruction caused by large fecalith formation within a blind loop 1 year after side-to-side anastomosis. This report highlights the importance of surgical technique selection and standardization in preventing BLS-related complications, providing valuable insights for clinical practice.

## Data Availability

The original contributions presented in the study are included in the article/supplementary material, further inquiries can be directed to the corresponding author.
